# Fracture risk in 85-year-old women: associations with volumetric bone mineral density, physical performance and falls

**DOI:** 10.1007/s11657-026-01777-8

**Published:** 2026-11-12

**Authors:** Axel Wihlborg, Kristina E. Åkesson, Paul Gerdhem

**Affiliations:** 1 Department of Clinical Science, Intervention and Technology, Karolinska Institutet, Stockholm, Sweden; 2 Department of Orthopedics, Karolinska University Hospital, Stockholm, Sweden; 3 Department of Clinical Science in Malmö, Lund University, Lund, Sweden; 4 Department of Orthopedics, Skåne University Hospital, Malmö, Sweden; 5 Department of Surgical Sciences, Uppsala University Hospital, Uppsala, Sweden; 6 Department of Orthopaedics and Hand Surgery, Uppsala University Hospital, Uppsala, Sweden

**Keywords:** Fracture risk, Frailty, pQCT, Falls, Physical performance test

## Abstract

***Summary*:**

This prospective study demonstrates associations of volumetric BMD (vBMD) by pQCT, areal BMD (aBMD) by DXA, physical performance (one-leg balance, gait speed, and knee extension force), and fall history with fracture risk in 85-year-old women. A failed one-leg balance test was associated with both hip and any fracture independent of femoral neck aBMD. Total radial vBMD was associated with any fracture. Gait speed and previous falls showed associations with fracture risk of similar magnitude as femoral neck aBMD. These findings suggest that functional performance measures may enhance fracture risk assessment in older women.

**Purpose:**

This prospective study evaluates associations between fracture risk and volumetric bone mineral density (vBMD) by peripheral quantitative computed tomography (pQCT), physical performance tests, and fall history, in comparison with femoral neck areal BMD (aBMD) by DXA, in 85-year-old women.

**Methods:**

In the Osteoporosis Prospective Risk Assessment (OPRA) study, 382 women aged 85 years were followed for a mean of 4.3 years. Participants underwent pQCT of the radius and tibia and DXA of the hip and lumbar spine, performed physical performance tests (one-leg standing balance, considered failed if balance could not be maintained for 5 s; gait speed, analyzed continuously; and knee extension force, measured by isometric dynamometry), and reported fall history. Cox proportional hazards models estimated fracture risk, adjusting for BMI, previous fracture, smoking, and use of vitamin D, glucocorticoids, and bisphosphonates.

**Results:**

During a mean follow-up of 4.3 years, 116 women (30%) sustained a fracture. Trabecular vBMD at the radius and tibia was associated with lower risk of any fracture (HR 0.68, 95% CI 0.55–0.85 and HR 0.69, 95% CI 0.55–0.86, respectively). Radial trabecular vBMD was also associated with lower hip fracture risk (HR 0.64, 95% CI 0.42–0.98). Femoral neck aBMD was associated with lower risk of any fracture (HR 0.75, 95% CI 0.62–0.90), but not hip fracture (HR 0.71, 95% CI 0.50–1.00). A failed balance test and low gait speed was associated with higher risk of any fracture (HR 1.55, 95% CI 1.04–2.29 and HR 1.22, 95% CI 1.01–1.48, respectively). In multivariate analyses, a failed balance test remained associated with any fracture (HR 1.70, 95% CI 1.12–2.56) and hip fracture (HR 2.30, 95% CI 1.05–5.02). Total radial vBMD remained associated with any fracture (HR 0.79, 95% CI 0.63–0.996).

**Conclusion:**

Volumetric BMD by pQCT was associated with fracture risk independent of commonly recognized clinical risk factors in 85-year-old women. Physical performance tests were associated with fracture risk in unadjusted analyses, and the one-leg balance test additionally remained associated after adjustment for clinical risk factors and femoral neck aBMD.

## Introduction

Identifying and quantifying risk factors for fracture is of great importance in improving fracture risk assessment. Standard methods of assessing fracture risk, such as FRAX (Fracture Risk Assessment Tool), integrate bone mineral density of the femoral neck (aBMD), assessed by dual-energy X-ray absorptiometry (DXA) [1, 2], with several validated clinical risk factors such as age [3, 4], body mass index (BMI) [5], smoking [6, 7], glucocorticoid use [8] and previous fracture [9]. While the correlation with fracture risk is well-supported, these factors do not fully encompass the complexity of fracture risk. In a large multi-cohort analysis, combining clinical risk factors with femoral neck aBMD increased the risk gradient for hip fracture only modestly compared with aBMD alone (from 3.7/SD to 4.2/SD at the age of 50 years), and even smaller increment was observed for other osteoporotic fractures [10]. This emphasizes the need to study additional risk factors and investigative methods.

Peripheral quantitative computed tomography (pQCT) measures three-dimensional volumetric bone mineral density (vBMD) and assesses geometric properties such as bone size. In addition, it also allows for separate evaluations of the trabecular and cortical components. High resolution pQCT (HR-pQCT) in addition assesses microarchitecture and estimated bone strength. Both imaging techniques have to some extent been associated with fracture risk independently of aBMD by DXA [11–16] suggesting that volumetric and geometric parameters provide additional information beyond areal BMD alone. Both HR-pQCT and conventional pQCT are primarily research tools with limited clinical availability. Although the evidence is more extensive for HR-pQCT, to our knowledge, no direct comparison between conventional pQCT and HR-pQCT for fracture prediction has been reported.

Reduced gait speed and impaired balance, assessed by one-leg standing time or tandem walk, have been associated with hip and other major osteoporotic fractures, independent of age, femoral neck aBMD, and other clinical risk factors, in large prospective cohorts, including a population-based cohort of 3028 women aged 75–80 in Sweden [17], and a community based cohort of 6007 adults with a mean age of 66 years in South Korea [18]. This is further supported by a recent review of 67 prospective studies [19]. In addition, a history of previous falls has been associated with an increased risk of any fracture independent of femoral neck aBMD, as shown in a prospective cohort study from the UK and confirmed in a meta-analysis of 46 cohorts [20, 21]. Physical performance and a history of falls are important fracture risk factors that are not captured by standard risk assessment tools such as FRAX. These factors may become increasingly relevant with advancing age in older adults, because of physical decline, frailty, and increased likelihood of falls [19, 22, 23].

We have previously shown, in the same cohort of women examined 10 years earlier at the age of 75, that a failed balance test (defined as the inability to maintain standing balance for 5 s on either leg) and reduced gait speed were associated with an increased 10-year risk of hip fracture independent of femoral neck aBMD [24]. A history of falls demonstrated stronger associations with fracture risk than the one-leg balance test and gait speed at older age (80–85 years) when compared to ages 75–80 [24]. Although aBMD, functional performance, and history of falls have been linked to fracture risk in older adults, their relative contributions in women aged  85 years remain unclear.

In this prospective study of women aged 85 years, we investigate the association of volumetric BMD measured by pQCT at the distal radius and tibia, functional performance tests, and history of falls in relation to fracture risk. We hypothesize that both radial and tibial volumetric BMD will be associated with fracture risk independent of commonly used clinical risk factors (body mass index, smoking, glucocorticoid use, vitamin D use, bisphosphonate use, and previous fracture). Furthermore, we hypothesize that measures of functional performance and history of falls may provide additional information on fracture risk beyond femoral neck and lumbar spine aBMD by DXA and clinical risk factors in this population of 85-year-old women.

## Materials and methods

The Osteoporosis Prospective Risk Assessment study (OPRA) was initiated in 1995 with the aim of studying risk factors for fracture and osteoporosis in women. Details of the study cohort including external validity has previously been published [24, 25]. Between 1995 and 1999 all women at the age of 75 in the city of Malmö, were invited to participate in the population-based study. In total, 1044 out of 1604 women responded to the invitation and extensive examinations were carried out regularly (every 5 years) at the Clinical and Molecular Osteoporosis Research Unit, Skåne University Hospital, Malmö, Sweden. Mortality data was continuously obtained from the national population files.

At the 10-year follow-up, 382 women at the age of 85 participated. Of the 662 women who did not attend, 307 had not survived to age 85. Of the remaining 355 women, illness was the most common reported reason for non-attendance. These women were part of the ongoing OPRA cohort and had participated in previous examinations since inclusion at the age of 75. In this study, these women are followed for incident fractures until November 2012 or death. Participants underwent peripheral quantitative computed tomography (pQCT), dual-energy X-ray absorptiometry (DXA), blood sampling, physical performance tests, and completed a self-assessment questionnaire at the 10-year follow-up.

Volumetric bone properties were assessed at the forearm and lower leg with pQCT (Stratec XCT-2000, Stratec Medizintechnik). The coefficient of variation (CV%) was 0.27 for the forearm phantom. Version 6.2 of the manufacturer software was used. Scans were acquired and analyzed according to standard protocols, with a voxel size of 0.5 mm. Bone segmentation was performed using contour mode 1, cortical mode 1, and peel mode 1, with a threshold of 180 mg/cm³ applied for trabecular and cortical separation. Measurements were performed at the distal (4%) and shaft (66%) site of the radial bone, and at the distal (4%) and shaft (38%) site of the tibial bone, in accordance with the Stratec XCT-2000 manufacturer protocol and standard pQCT methodology [16]. Trabecular bone properties are reported as volumetric trabecular bone mineral density (vBMD), volumetric total bone mineral content (vBMC) and total vBMD at the distal site. Cortical bone properties are reported as total vBMC and cortical vBMD at the shaft site. Geometric properties were assessed by cross-sectional area (CSA) at the distal site and cortical thickness at the shaft site.

Areal bone properties were assessed at the hip and lumbar spine with DXA (Lunar DPX-L, Lunar Corporation). The coefficient of variation (CV%) for DXA measurements in this cohort has previously been reported as 1.46% for femoral neck, 1.10% for total hip, and 3.44% for lumbar spine [26]. Areal bone mineral density (aBMD) of the femoral neck and the L1-L2 segment of the spine is reported due to more degenerative changes in the lower segments of the spine [27].

Physical performance tests (knee extension force, standing balance and gait speed) were carried out as previously described [24]. The best out of 3 attempts was registered. Knee extension force was determined by isometric muscle contraction for 5 s with 90 degrees flexion of the knee, measured with a computerized dynamometer (Biodex Medical Systems, v4.5.0, Biodex Corporation). Standing balance was determined by how long participants could maintain balance standing on either leg. Not managing to maintain balance for 5 s was considered a failed balance test. The 5-s threshold was derived from observations in this cohort at age 75. Participants unable to maintain balance for 5 s generally could not maintain postural stability, and very few were able to reach 10 s. This same threshold has been applied in a previous study of the cohort [24]. Gait speed was measured in m/s and determined by the time needed to walk 15 meters, turn 180 degrees, and walk 15 meters back. The recorded speed therefore reflects both straight-line walking and turning ability. Participants were encouraged to walk as fast as they could. Although coefficients of variation (CV) data were not available, the reliability of knee extension force, one-leg balance and gait speed has been documented in previous studies [28–30].

Fractures were registered through radiology files from the city hospital and cross-checked with questionnaire information. The registration can be regarded as nearly complete with less than 3% missed cases [24]. All fractures in the study were the result of low-energy trauma, determined through review of radiology referrals and medical records. The first occurrence of a specific fracture type was registered. Multiple fractures refer to two or more fractures occurring during follow-up, either at the same time or consecutively. Fractures were analyzed individually and categorized as either any fracture or major osteoporotic fracture. Major osteoporotic fractures included hip, distal forearm, proximal humerus, and clinical vertebral fractures. For the fracture-specific outcomes, each woman could contribute an event to more than one fracture specific outcome if she sustained fractures at different sites. For the composite outcomes (any fracture and major osteoporotic fracture), only the time to the first event was used.

History of a fall refers to at least one self-reported fall within one year prior to the investigation point and history of multiple falls refers to the occurrence of two or more falls within the same period. Fall history, history of previous fracture, and alcohol-, smoking, bisphosphonate-, vitamin D-, and glucocorticoid (cortisone) use were obtained through a self-assessment questionnaire. The questionnaire was administered at each examination visit and has been used consistently throughout the OPRA study, although it has not been formally validated. The questionnaire also collected additional information on lifestyle and health not used in the present analyses.

Cox proportional-hazard regression was used to determine univariate hazard ratios (95% CI). Participants who died without sustaining a fracture were treated as right-censoring events in the Cox models. Time-to-event was defined as time to fracture, death, or end of follow-up. In the multivariate analyses, covariates included body mass index (BMI), previous fracture, smoking habits, bisphosphonate use, vitamin D use, and glucocorticoid use. For analyses of physical performance measures, models were additionally adjusted for femoral neck aBMD (vertebral aBMD for vertebral fracture). Continuous data were logarithmized in case of non-normal distribution. Model assumptions were assessed by evaluating proportional hazards and by visual inspection of residual plots. Knee extension force, gait speed, DXA and pQCT data were standardized to allow for comparisons between variables.

Informed consent was obtained from all participants. The study was approved by the Ethics committee of Lund University (LU 200/95, 2014/804).

### Ethical approval

All procedures performed in studies involving human participants were in accordance with the ethical standards of the institutional and national research committee, and with the 1964 Helsinki declaration and its later amendments or comparable ethical standards

## Results

Mean follow-up from age 85 years was 4.3 years (0.1–6.8) and mortality was 28% (106 out of 382 women). Baseline characteristics are shown in Table 1. The mean femoral neck T-score was –2.4 (SD 1.1). The mean femoral neck Z-score was −0.00 (SD 1.0), indicating that bone mineral density in this cohort was similar to age-matched reference. Baseline characteristics of women with and without any fracture are shown in Table 2. Women who experienced a fracture had significantly lower femoral neck aBMD (*p* = 0.011, Table 2). A greater proportion of women who experienced a fracture failed the one-leg balance test (69% vs. 59%), although this difference was not statistically significant (*p* = 0.066).

**Table 1:** Baseline characteristics of the 382 women

Baseline variable	*n*: 382
Continuous variables	Mean (SD)
Age (years)	85.2 (0.1)
BMI (kg/m²)	25.5 (4.0)
Femoral neck BMD (g/cm^2^)	0.690 (0.136)
Femoral neck T-score	–2.4 (1.1)
Femoral neck Z-score	–0.00 (1.0)
Lumbar spine BMD L1-L2 (g/cm^2^)	0.910 (0.201)
Lumbar spine T-score L1-L2	–2.0 (1.7)
Gait speed (m/s)	1.1 (0.3)
Knee extension force (Nms)	218 (64)
Categorical variables	Number (%)
Failed balance test	237 (62)
History of a fall^a^	177 (46)
History of multiple falls^b^	98 (26)
History of any fracture	206 (54)
Smoking^c^	22 (6)
Alcohol use >2 units/day^d^	21 (5)
Bisphosphonate use^c^	12 (3)
Vitamin D use^c^	23 (6)
Glucocorticoid use^c^	49 (13)

Continuous variables presented as mean (SD) and categorical variables as number of women (%)

*SD* Standard deviation, *BMI* Body mass index, *BMD* Bone mineral density

^a^Reported at least one fall within 1 year prior to baseline

^b^Reported two or more falls within 1 year prior to baseline

^c^Current or previous use (smoking, glucocorticoid); current use (Vitamin D, bisphosphonates)

^d^Alcohol consumption corresponding to more than 2 units per day on average

**Table 2:** Baseline characteristics according to fracture status at follow-up

Baseline variable	No fracture *n*:266	Fracture *n*:116	p-value
Continuous variables	Mean (SD)	Mean (SD)	
BMI (kg/m²)	25.6 (4.0)	25.2 (4.1)	0.484
Femoral neck BMD (g/cm²)	0.702 (0.136)	0.662 (0.133)	0.011
Gait speed (m/s)	1.1 (0.3)	1.1 (0.3)	0.124
Knee extension force (Nms)	221 (64)	211 (62)	0.177
Categorical variables	Number (%)	Number (%)	
Failed balance test	157 (59)	80 (69)	0.066
Fall last year	120 (45)	57 (49)	0.468
Multiple falls	63 (24)	35 (30)	0.182

Fracture refers to any fracture sustained during the follow-up period. Continuous variables presented as mean (SD) and categorical variables as number of women (%)

During follow-up, 116 women (30%) sustained at least one fracture, and 41 women (11%) had two or more fractures (multiple fractures). A major osteoporotic fracture occurred in 99 women (26%); 34 hip, 44 clinical vertebral, 24 distal forearm and 15 proximal humerus fractures. During follow-up, 9 women sustained concurrent fractures at two sites on at least one occasion.

### pQCT and volumetric BMD

Hazard ratios (HR) for fracture risk for pQCT and DXA variables in the univariate model are shown in Table 3. Higher trabecular vBMD of both the radial and tibial bones was associated with a lower risk of any fracture (HR 0.68, 95% CI 0.55–0.85 and HR 0.69, 95% CI 0.55–0.86, respectively) and major osteoporotic fractures. Higher trabecular vBMD of the radial bone was moreover associated with a lower risk of hip fracture (HR 0.64, 95% CI 0.42–0.98). Higher cortical vBMD of the radial bone was associated with a lower risk of any fracture and of major osteoporotic fractures, as was higher cortical thickness at both sites. However, cortical vBMD of the tibial bone was not associated with fracture risk (Table 3).

**Table 3:** Unadjusted univariate hazard ratios for volumetric bone mineral density (vBMD) and geometric variables by pQCT, and areal bone mineral density (aBMD) by DXA

Fracture	Any *n*: 116 HR (95% CI)	Major osteoporotic *n*: 99 HR (95% CI)	Hip *n:* 34 HR (95% CI)	Clinical vertebral *n*: 44 HR (95% CI)	Distal forearm *n*: 24 HR (95% CI)	Proximal humerus *n*: 15 HR (95% CI)
PQCT Radial bone						
vBMD total 4%	0.72 (0.58–0.90)	0.77 (0.61–0.97)	0.70 (0.47–1.05)	0.95 (0.69–1.32)	0.56 (0.34–0.93)	0.70 (0.38–1.28)
vBMD trabecular 4%	0.68 (0.55–0.85)	0.70 (0.55–0.89)	0.64 (0.42–0.98)	0.87 (0.63–1.22)	0.63 (0.39–1.02)	0.53 (0.28–1.02)
vBMD cortical 66%	0.73 (0.60–0.89)	0.71 (0.57–0.88)	0.84 (0.59–1.19)	0.79 (0.58–1.09)	0.75 (0.50–1.12)	0.65 (0.40–1.06)
Cortical thickness 66%	0.73 (0.59–0.90)	0.71 (0.56–0.89)	0.71 (0.48–1.04)	0.86 (0.62–1.20)	0.62 (0.40–0.98)	0.58 (0.33–1.03)
CSA 4%	1.09 (0.89–1.33)	1.03 (0.83–1.28)	0.99 (0.69–1.42)	1.05 (0.77–1.43)	1.21 (0.80–1.82)	0.98 (0.58–1.65)
PQCT Tibial bone						
vBMD total 4%	0.66 (0.53–0.82)	0.67 (0.52–0.85)	0.82 (0.54–1.25)	0.81 (0.57–1.15)	0.75 (0.47–1.18)	0.55 (0.31–0.98)
vBMD trabecular 4%	0.69 (0.55–0.86)	0.70 (0.55–0.89)	0.78 (0.51–1.20)	0.89 (0.63–1.26)	0.71 (0.45–1.12)	0.60 (0.34–1.08)
vBMD cortical 38%	0.87 (0.71–1.07)	0.85 (0.68–1.06)	0.99 (0.66–1.48)	0.89 (0.64–1.24)	1.01 (0.65–1.57)	0.79 (0.48–1.30)
Cortical thickness 38%	0.73 (0.60–0.90)	0.76 (0.61–0.94)	0.85 (0.57–1.26)	0.90 (0.65–1.26)	0.73 (0.48–1.10)	0.70 (0.42–1.18)
CSA 4%	1.20 (0.98–1.47)	1.23 (0.99–1.54)	0.89 (0.60–1.32)	1.40 (1.00–1.96)	1.00 (0.65–1.54)	1.28 (0.76–2.15)
DXA						
BMD femoral neck	0.75 (0.62–0.90)	0.74 (0.60–0.91)	0.71 (0.50–1.00)	0.84 (0.61–1.15)	0.75 (0.49–1.15)	0.72 (0.43–1.23)
BMD L1-L2	0.86 (0.71–1.04)	0.78 (0.63–0.97)	0.98 (0.70–1.38)	0.71 (0.52–0.97)	0.92 (0.61–1.38)	0.63 (0.37–1.09)

Fracture hazard ratios are shown for each standard deviation increase of the variable

*CSA* Cross sectional area

### DXA and aBMD

Higher aBMD at the femoral neck was associated with lower risk of any fracture (HR 0.75, 95% CI 0.62–0.90) and major osteoporotic fractures, while higher vertebral aBMD was associated with a lower risk of major osteoporotic fractures and clinical vertebral fracture. Neither femoral neck nor vertebral aBMD was associated with hip fracture risk (Table 3).

The hazard ratios for pQCT and DXA variables in the multivariate model are shown in Table 4. Of the pQCT variables, only total vBMD of the radial bone remained significantly associated with fracture risk after adjustment for covariates (HR 0.79, 95% CI 0.63–0.996).

**Table 4:** Adjusted multivariate fracture hazard ratios for volumetric bone mineral density (vBMD) and geometric variables by pQCT, and areal bone mineral density (aBMD) by DXA

Fracture	Any HR (95% CI)	Major osteoporotic HR (95% CI)	Hip HR (95% CI)	Clinical vertebral HR (95% CI)	Distal forearm HR (95% CI)	Proximal humerus HR (95% CI)
PQCT Radial bone						
vBMD total 4%	0.79 (0.63–1.00)^a^	0.87 (0.68–1.10)	0.78 (0.52–1.18)	1.08 (0.77–1.52)	0.63 (0.38–1.04)	0.66 (0.32–1.35)
vBMD trabecular 4%	0.82 (0.65–1.03)	0.87 (0.68–1.11)	0.74 (0.48–1.13)	1.09 (0.77–1.54)	0.73 (0.44–1.21)	0.61 (0.30–1.24)
vBMD cortical 66%	0.83 (0.67–1.01)	0.81 (0.65–1.00)	0.95 (0.67–1.34)	0.90 (0.66–1.24)	0.84 (0.57–1.25)	0.70 (0.42–1.15)
Cortical thickness 66%	0.82 (0.66–1.02)	0.80 (0.63–1.01)	0.81 (0.55–1.19)	0.99 (0.71–1.39)	0.68 (0.43–1.09)	0.61 (0.34–1.11)
CSA 4%	1.00 (0.82–1.21)	0.94 (0.76–1.16)	0.91 (0.64–1.30)	0.97 (0.71–1.31)	1.15 (0.76–1.75)	0.88 (0.52–1.49)
PQCT Tibial bone						
vBMD total 4%	0.87 (0.66–1.13)	0.95 (0.71–1.27)	1.14 (0.71–1.85)	1.13 (0.73–1.73)	0.98 (0.57–1.66)	0.61 (0.28–1.34)
vBMD trabecular 4%	0.90 (0.69–1.16)	0.97 (0.74–1.28)	1.05 (0.65–1.69)	1.28 (0.84–1.96)	0.88 (0.53–1.49)	0.74 (0.34–1.59)
vBMD cortical 38%	0.97 (0.78–1.20)	0.94 (0.75–1.18)	1.10 (0.72–1.66)	0.97 (0.69–1.36)	1.09 (0.70–1.69)	0.89 (0.52–1.52)
Cortical thickness 38%	0.86 (0.69–1.07)	0.91 (0.71–1.16)	1.03 (0.66–1.61)	1.09 (0.74–1.61)	0.82 (0.52–1.30)	0.78 (0.43–1.42)
CSA 4%	1.07 (0.87–1.32)	1.09 (0.87–1.37)	0.77 (0.51–1.17)	1.32 (0.92–1.88)	0.90 (0.58–1.42)	1.11 (0.64–1.93)
DXA						
aBMD femoral neck	0.83 (0.67–1.03)	0.86 (0.68–1.09)	0.78 (0.52–1.16)	0.97 (0.67–1.41)	0.88 (0.55–1.42)	0.77 (0.40–1.46)
aBMD L1-L2	0.97 (0.78–1.21)	0.90 (0.70–1.15)	1.14 (0.77–1.67)	0.74 (0.52–1.08)	1.14 (0.72–1.81)	0.53 (0.27–1.06)

Fracture hazard ratios are shown for each standard deviation increase of the variable. Covariates in the multivariate analysis: BMI, history of fracture, smoking habits, bisphosphonate-, Vitamin D- and glucocorticoid use

*CSA* Cross sectional area

^a^ 0.79 (0.63–0.996)

### Physical performance tests and history of falls

Table 5 shows fracture hazard ratios for physical performance tests and history of falls. A failed balance test and lower gait speed were associated with a higher risk of any fracture (HR 1.55, 95% CI 1.04–2.29 and HR 1.22 (1.01–1.48), respectively). Low knee extension force was associated with a higher distal forearm fracture risk. History of multiple falls was associated with a higher risk of major osteoporotic fractures and clinical vertebral fractures. After adjustment for covariates, including aBMD, a failed balance test remained significantly associated with a higher risk of any fracture (HR 1.70, 95% CI 1.12–2.56) and hip fracture (HR 2.30, 95% CI 1.05–5.02) (Table 5).

**Table 5:** Unadjusted (univariate) and adjusted (multivariate) fracture hazard ratios for tests of physical performance and self-reported history of falls

Fracture	Any HR (95% CI)	Major osteoporotic HR (95% CI)	Hip HR (95% CI)	Clinical vertebral HR (95% CI)	Distal forearm HR (95% CI)	Proximal humerus HR (95% CI)
Univariate						
Knee extension force ^a^	1.18 (0.97–1.45)	1.18 (0.95–1.46)	1.21 (0.82–1.77)	1.19 (0.84–1.69)	1.64 (1.07–2.51)	0.83 (0.52–1.34)
Failed Balance test	1.55 (1.04–2.29)	1.29 (0.85–1.95)	1.93 (0.90–4.14)	1.38 (0.73–2.60)	0.90 (0.40–2.03)	0.76 (0.27–2.08)
Gait speed ^a^	1.22 (1.01–1.48)	1.14 (0.93–1.40)	1.26 (0.88–1.80)	1.32 (0.97–1.81)	0.92 (0.63–1.36)	1.00 (0.61–1.65)
History of a fall	1.16 (0.81–1.67)	1.18 (0.80–1.75)	1.37 (0.70–2.68)	1.07 (0.59–1.94)	1.01 (0.45–2.25)	1.40 (0.51–3.86)
History of multiple falls	1.36 (0.91–2.01)	1.60 (1.05–2.43)	1.29 (0.62–2.70)	1.92 (1.04–3.52)	1.27 (0.52–3.05)	0.77 (0.22–2.72)
Multivariate						
Knee extension force ^a^	1.20 (0.97–1.49)	1.19 (0.93–1.49)	1.16 (0.78–1.75)	1.12 (0.76–1.67)	1.67 (1.04–2.70)	0.72 (0.42–1.27)
Failed Balance test	1.70 (1.12–2.56)	1.48 (0.95–2.28)	2.30 (1.05–5.02)	1.37 (0.72–2.61)	1.04 (0.43–2.50)	0.66 (0.24–1.86)
Gait speed ^a^	1.18 (0.95–1.45)	1.12 (0.90–1.39)	1.35 (0.91–2.04)	1.19 (0.87–1.64)	0.90 (0.63–1.30)	0.87 (0.56–1.33)
History of fall	0.94 (0.64–1.37)	0.92 (0.61–1.39)	1.32 (0.66–2.64)	0.87 (0.47–1.58)	0.92 (0.39–2.19)	1.27 (0.44–3.70)
History of multiple falls	1.04 (0.69–1.58)	1.23 (0.80–1.91)	1.11 (0.52–2.36)	1.57 (0.84–2.93)	1.05 (0.40–2.75)	0.61 (0.17–2.28)

Hazard ratios are presented per standard deviation decrease for Knee extension force and Gait speed. Other variables are categorical and presented as group comparisons. Covariates in the multivariate analysis: femoral neck aBMD (L1-L2 aBMD for vertebral fracture), BMI, history of fracture, smoking habits, bisphosphonate-, Vitamin D- and glucocorticoid use

^a^Fracture hazard ratio presented for each standard deviation decrease of the variable

Hazard ratios for the covariates used in this study (BMI, history of any fracture, smoking habits, bisphosphonate use, Vitamin D use and glucocorticoid use) are shown in Table 6. History of any fracture was strongly associated with fracture risk, with fracture hazard ratios exceeding those of the other variables studied.

**Table 6:** Unadjusted univariate hazard ratios for selected covariates in Tables 4, 5

Fracture	Any HR (95% CI)	Major osteoporotic HR (95% CI)	Hip HR (95% CI)	Clinical vertebral HR (95% CI)	Distal forearm HR (95% CI)	Proximal humerus HR (95% CI)
History of any fracture	4.50 (2.85–7.11)	6.20 (3.58–10.75)	3.43 (1.49–7.87)	9.53 (3.41–26.64)	3.35 (1.25–8.97)	12.52 (1.65–95.21)
BMI	0.90 (0.74–1.09)	0.82 (0.66–1.01)	0.93 (0.66–1.30)	0.89 (0.66–1.21)	0.71 (0.46–1.10)	1.01 (0.61–1.68)
Smoking habits	0.73 (0.30–1.80)	0.70 (0.26–1.91)	0.51 (0.07–3.74)	0.84 (0.20–3.48)	0.76 (0.10–5.66)	1.33 (0.18–10.12)
Bisphosphonate use	4.38 (2.20–8.70)	4.11 (1.98–8.53)	1.11 (0.15–8.12)	3.66 (1.31–10.27)	1.66 (0.22–12.30)	15.09 (4.04–56.31)
Vitamin D use	2.82 (1.58–5.04)	2.95 (1.61–5.41)	0.48 (0.07–3.49)	3.41 (1.52–7.65)	1.54 (0.36–6.56)	4.35 (1.23–15.42)
Glucocorticoid use	1.18 (0.71–1.98)	1.31 (0.77–2.24)	0.63 (0.19–2.05)	1.79 (0.86–3.73)	1.35 (0.46–3.94)	2.55 (0.81–8.00)

Hazard ratios are presented per standard deviation increase for BMI and as categorical comparisons for all other variables

## Discussion

The findings in this population-based prospective study of 85-year-old women suggest that low total radial vBMD and a failed one-leg balance test provide additional information on fracture risk beyond what is captured by clinical risk factors (body mass index, previous fracture, smoking habits, bisphosphonate use, vitamin D use, and glucocorticoid use). Low pQCT variables of both the radial and tibial bone (cortical thickness, total and trabecular vBMD), as well as reduced gait speed, demonstrated risk associations of similar magnitude as femoral neck and lumbar spine aBMD. Multiple falls showed associations of larger magnitude with major osteoporotic and vertebral fracture in unadjusted analyses. However, prior fracture showed the strongest association with subsequent fractures. After adjustment for the clinical risk factors listed above, total radial vBMD and one-leg balance test remained significantly associated with fracture risk, whereas femoral neck aBMD did not remain significant. For the one-leg balance test, this association was additionally independent of femoral neck aBMD, suggesting that postural balance captures an aspect of fracture risk not fully reflected by aBMD by DXA. Whether radial vBMD provides information on fracture risk independent of aBMD could not be assessed in the present study, as the pQCT models were not adjusted for aBMD.

Low trabecular vBMD at the distal radius was the only bone parameter significantly associated with both overall fracture risk and hip fracture risk in the unadjusted model, while total vBMD at the distal radius was the only parameter that remained significant after adjustment in the multivariate model. These findings are consistent with previous prospective studies demonstrating associations between low vBMD at the distal radius and fracture risk, predominantly using HR-pQCT, with HR 1.4–1.9 per SD decrease [11, 12, 14]. Associations between pQCT measures at the radius and tibia and incident fracture have also been reported with HR of 1.6–1.9 per SD decrease for radial cortical parameters [16]. Although most prior studies have used HR-pQCT rather than conventional pQCT, our findings indicate that conventional pQCT-derived measures of volumetric BMD at the distal radius were also associated with fracture risk in this population. No direct comparison between conventional pQCT and HR-pQCT was made in this study. The upper 95% confidence interval for total radial vBMD in the multivariate model was close to unity (0.996), and as several pQCT variables were tested, this finding should be interpreted with caution and warrants confirmation in larger cohorts.

The incidence of hip fracture increases with advancing age, reflecting the combined effects of declining bone strength, increased fall risk, and age-related frailty [4]. However, in this cohort of women at age 85 and above, femoral neck aBMD was not associated with hip fracture risk, and no associations between femoral neck or lumbar spine aBMD and fracture risk remained after multivariate adjustment. In agreement with previous findings [11–15], our results suggest that volumetric BMD assessed by pQCT was associated with fracture risk in this population of older women. However, pQCT remains primarily a research tool with limited utility in routine clinical practice, while DXA is widely available in clinical settings.

A failed one-leg balance test was associated with fracture risk independent of femoral neck aBMD and was the only variable in this study with independent association with hip fracture risk. In addition, experiencing multiple falls was associated with sustaining a major osteoporotic fracture in the unadjusted analysis. This is in line with previous findings that postural stability tests are important factors in assessing fracture risk [19, 20, 24]. In the SUPERB cohort of women aged 75–80 years, a failed one-leg standing test below 10 s was associated with a 76% increased risk of major osteoporotic fracture and a threefold increased risk of hip fracture, independent of FRAX clinical risk factors and femoral neck aBMD [17]. Our findings extend these observations to women aged 85 years. This suggests that postural balance may play an important role in fracture risk, independent of bone density, particularly with advancing age. The one-leg standing balance test is simple, inexpensive, and easy to perform in clinical practice.

We have previously reported an association between reduced gait speed and hip fracture risk at the age of 75 [24]. Although an association between gait speed and fracture risk in general was observed in the present study, no association was found with hip fracture risk. Reduced gait speed has been consistently associated with increased fracture risk in prospective cohorts, with all studies in a recent scoping review reporting significant associations between slower gait speed and major osteoporotic fracture [19]. Low knee extension force was associated with distal forearm fracture risk in the present study, an association not observed in our previous study [24]. This may indicate that the influence of lower extremity muscle strength on fall risk increases with advancing age. However, measuring knee extension force requires a computerized dynamometer, which is less practical for routine clinical use compared to a one-leg balance test or gait speed test. Integrating these functional assessments could offer a more effective approach to assessing fracture risk in older populations.

Previous findings regarding the associations between femoral neck aBMD, clinical risk factors and fracture risk in elderly populations have been contradictory, with reports indicating both stronger associations with major osteoporotic fractures and weaker associations with hip fracture risk for femoral neck aBMD [1,31,32]. While clinical risk factors have been shown to outperform femoral neck aBMD in predicting osteoporotic fractures with advancing age, this does not appear to apply to hip fractures [10]. Conversely, in a large population of elderly women in Sweden, femoral neck aBMD was found to have a stronger influence on fracture risk than clinical risk factors [33]. These discrepancies may partly reflect differences in age ranges studied, fracture ascertainment methods, and the specific clinical risk factors included in each model.

The strong association between a history of fracture and subsequent fracture risk is well known, based on a large meta-analysis spanning multiple cohorts and a wide range of ages [9]. Our results suggest a 3 to 10-fold higher fracture risk compared with DXA and pQCT measurements and physical performance tests. Given the strength of this association, history of fracture accounts for a substantial part of the explained variation in fracture risk. However, of the 116 women who sustained an incident fracture during follow-up, 23 (20%) had no prior fracture history, and additional assessments may therefore help identify high-risk individuals before their first fracture.

The increasing proportion of women who have sustained fractures over time may partly account for part of the relatively high fracture ratios observed. However, prior studies have shown that the risk ratios for previous fractures decrease with advancing age and over time [9].

Bisphosphonate and vitamin D use were included as covariates and showed elevated hazard ratios for some fractures (Table 6), but with wide confidence intervals due to the small number of users. These associations are likely explained by confounding by indication, as women with prior fractures were more frequently prescribed these treatments rather than reflecting independent associations with fracture risk. Bisphosphonates were the only osteoporosis-specific medication included in this study. The low proportion of women on bisphosphonate treatment (3%) despite a high prevalence of prior fracture (54%) reflects the well-documented treatment gap in osteoporosis management in Sweden, where only 8–17% of older women with a previous fragility fracture receive osteoporosis medication [34].

### Strengths and limitations

This study has several limitations. The observational design limits our ability to interpret causality, and the study population may not be representative of all older women. The representativeness of the OPRA cohort has been described previously, participants had lower mortality and slightly higher fracture rates at baseline compared to non-participants [25]. Furthermore, women attending the 10-year follow-up were less frail at baseline than those who had not survived to age 85 or were alive but did not participate [35]. This healthy survivor effect should be considered when interpreting the findings. The mean follow-up time of 4.3 years may limit the ability to observe long-term outcomes or trends. However, the relatively short follow-up reflects the advanced age of the participants (85 years), where mortality rates are high. The mortality rate could also introduce bias in assessing longer term fracture risk. Additionally, the relatively small number of fracture events for certain outcomes, particularly hip fracture (*n* = 34), may reduce the statistical power to detect associations, including between gait speed and hip fracture. One-leg standing time could alternatively be analyzed as a continuous variable but given the high proportion of women unable to maintain balance for 5 s in this cohort (Table 1), a binary classification was considered most clinically interpretable. A validated cut-off for women aged  85 years is to our knowledge not available. Other medications with potential effects on fracture risk were not recorded and may represent a source of confounding. The additive effect of multiple risk factors was not assessed in the present study and may be the subject of future studies.

This study also has several strengths. The study cohort of older women, 85 years of age, represents a high-risk population group for osteoporosis and fractures, with clear benefits from preventive measures. A comprehensive range of assessments were conducted, including pQCT, DXA and physical performance tests. Although overall functional assessments such as the Short Physical Performance Battery (SPPB) are widely used, the present study examined individual tests according to the OPRA protocol. Additionally, the population-based prospective design enhances the external validity.

In summary, our study shows that radial pQCT and one-leg balance testing were associated with fracture risk independent of commonly used clinical risk factors in 85-year-old women. The one-leg balance test additionally showed independence from femoral neck areal BMD measured by DXA. Gait speed was associated with fracture risk in unadjusted analyses, and knee extension force was associated with distal forearm fracture risk. Simple physical performance tests, particularly the one-leg balance test, may be practical and clinically useful measures for fracture risk assessment.  Integrating physical performance measures with bone density assessment may enhance fracture risk assessment in older women.

## Data Availability

The data used to support the findings of this study are available from the corresponding author upon request.

## References

[1] Johnell O et al (2005) Predictive value of BMD for hip and other fractures. J Bone Miner Res 20:1185–1194. 10.1359/JBMR.050304 15940371

[2] Cranney A, Jamal S, Tsang J, Josse R, Leslie W (2007) Low bone mineral density and fracture burden in postmenopausal women. Can Med Assoc J 177:575–580. 10.1503/cmaj.070234 PMC1963365 17846439

[3] Kanis J et al (2021) The effect on subsequent fracture risk of age, sex, and prior fracture site by recency of prior fracture. Osteoporos Int 32:1547–1555. 10.1007/s00198-020-05803-4 33537845

[4] Ensrud K (2013) Epidemiology of fracture risk with advancing age. J Gerontol Ser A-Biol Sci Med Sci 68:1236–1242. 10.1093/gerona/glt092 23833201

[5] De Laet C et al (2005) Body mass index as a predictor of fracture risk: a meta-analysis. Osteoporos Int 16:1330–1338. 10.1007/s00198-005-1863-y 15928804

[6] Thorin MH, Wihlborg A, Åkesson K, Gerdhem P (2016) Smoking, smoking cessation, and fracture risk in elderly women followed for 10 years. Osteoporos Int 27:249–255. 10.1007/s00198-015-3290-z 26302684

[7] Kanis J et al (2005) Smoking and fracture risk: a meta-analysis. Osteoporos Int 16:155–162. 10.1007/s00198-004-1640-3 15175845

[8] Kanis J et al (2004) A meta-analysis of prior corticosteroid use and fracture risk. J Bone Miner Res 19:893–899. 10.1359/JBMR.040134 15125788

[9] Kanis J et al (2023) Previous fracture and subsequent fracture risk: a meta-analysis to update FRAX. Osteoporos Int 34:2027–2045. 10.1007/s00198-023-06870-z PMC7615305 37566158

[10] Kanis J et al (2007) The use of clinical risk factors enhances the performance of BMD in the prediction of hip and osteoporotic fractures in men and women. Osteoporos Int 18(no. 8):1033–1046. 10.1007/s00198-007-0343-y 17323110

[11] Sornay-Rendu E, Boutroy S, Duboeuf F, Chapurlat R (2017) Bone microarchitecture assessed by HR-pQCT as predictor of fracture risk in postmenopausal women: the OFELY study. J Bone Miner Res 32:1243–1251. 10.1002/jbmr.3105 28276092

[12] Biver E, Durosier-Izart C, Chevalley T, van Rietbergen B, Rizzoli R, Ferrari S (2018) Evaluation of radius microstructure and areal bone mineral density improves fracture prediction in postmenopausal women. J Bone Miner Res 33:328–337. 10.1002/jbmr.3299 28960489

[13] Burt L, Manske S, Hanley D, Boyd S (2018) Lower bone density, impaired microarchitecture, and strength predict future fragility fracture in postmenopausal women: 5-Year follow-up of the calgary CaMos cohort. J Bone Miner Res 33:589–597. 10.1002/jbmr.3347 29363165

[14] Samelson E et al (2019) Cortical and trabecular bone microarchitecture as an independent predictor of incident fracture risk in older women and men in the Bone Microarchitecture International Consortium (BoMIC): a prospective study. Lancet Diab Endocrinol 7:34–43. 10.1016/S2213-8587(18)30308-5 PMC6354581 30503163

[15] Engelke K et al (2008) Clinical use of quantitative computed tomography and peripheral quantitative computed tomography in the management of osteoporosis in adults: the 2007 ISCD Official Positions. J Clin Densitom 11:123–162. 10.1016/j.jocd.2007.12.010 18442757

[16] Dennison EM, Jameson KA, Edwards MH, Denison HJ, Aihie Sayer A, Cooper C (2014) Peripheral quantitative computed tomography measures are associated with adult fracture risk: the Hertfordshire Cohort Study. Bone 64:13–17. 10.1016/j.bone.2014.03.040 24680720

[17] Larsson BAM et al (2022) One leg standing time predicts fracture risk in older women independent of clinical risk factors and BMD. Osteoporos Int 33(no. 1):185–194. 10.1007/s00198-021-06039-6 PMC8758616 34498096

[18] Lim Y, Ha J, Yoon KH, Baek KH, Kang MI (2021) Measures of physical performance as a predictor of fracture risk independent of BMD: the Chungju metabolic disease cohort study. Bone 145:115878. 10.1016/j.bone.2021.115878 33571697

[19] Vendrami C et al (2024) Muscle parameters in fragility fracture risk prediction in older adults: a scoping review. J Cachexia Sarcopenia Muscle 15(no. 2):477–500. 10.1002/jcsm.13418 PMC10995267 38284511

[20] Edwards M et al (2013) Clinical risk factors, bone density and fall history in the prediction of incident fracture among men and women. Bone 52:541–547. 10.1016/j.bone.2012.11.006 PMC3654628 23159464

[21] Vandenput L et al (2024) A meta-analysis of previous falls and subsequent fracture risk in cohort studies. Osteoporos Int 35:469–494. 10.1007/s00198-023-07012-1 38228807

[22] Cummings SR, Melton LJ (2002) Epidemiology and outcomes of osteoporotic fractures. Lancet 359(no. 9319):1761–1767. 10.1016/s0140-6736(02)08657-9 12049882

[23] Bartosch P, Kristensson J, McGuigan F, Akesson K (2020) Frailty and prediction of recurrent falls over 10 years in a community cohort of 75-year-old women. Aging Clin Exp Res 32(no. 11):2241–2250. 10.1007/s40520-019-01467-1 PMC7591409 31939201

[24] Wihlborg A, Englund M, Åkesson K, Gerdhem P (2015) Fracture predictive ability of physical performance tests and history of falls in elderly women: a 10-year prospective study. Osteoporos Int 26:2101–2109. 10.1007/s00198-015-3106-1 25832178

[25] Wihlborg A, Åkesson K, Gerdhem P (2014) External validity of a population-based study on osteoporosis and fracture. Acta Orthop 85:433–437. 10.3109/17453674.2014.920987 PMC4105777 24847791

[26] Lenora J, Akesson K, Gerdhem P (2010) Effect of precision on longitudinal follow-up of bone mineral density measurements in elderly women and men. J Clin Densitom 13(no. 4):407–412. 10.1016/j.jocd.2010.04.004 20605500

[27] Tenne M, McGuigan F, Besjakov J, Gerdhem P, Åkesson K (2013) Degenerative changes at the lumbar spine-implications for bone mineral density measurement in elderly women. Osteoporos Int 24(no. 4):1419–1428. 10.1007/s00198-012-2048-0 22733092

[28] Schroeder ET et al (2007) Reliability of maximal voluntary muscle strength and power testing in older men. J Gerontol A Biol Sci Med Sci 62(no. 5):543–549. 10.1093/gerona/62.5.543 17522360

[29] Springer BA, Marin R, Cyhan T, Roberts H, Gill NW (2007) Normative values for the unipedal stance test with eyes open and closed. J Geriatr Phys Ther 30(no. 1):8–15. 10.1519/00139143-200704000-00003 19839175

[30] Studenski S et al (2011) Gait speed and survival in older adults. Jama 305(no. 1):50–58. 10.1001/jama.2010.1923 PMC3080184 21205966

[31] Looker A (2013) Femur neck bone mineral density and fracture risk by age, sex, and race or Hispanic origin in older US adults from NHANES III. Arch Osteoporos 8:141. 10.1007/s11657-013-0141-4. 23715737

[32] Siris E et al (2006) The effect of age and bone mineral density on the absolute, excess, and relative risk of fracture in postmenopausal women aged 50-99: results from the National Osteoporosis Risk Assessment (NORA). Osteoporos Int 17(no. 4):565–574. 10.1007/s00198-005-0027-4 16392027

[33] Lauppe R et al (2019) Differing impact of clinical factors on the risk of fracture in younger and older women in the general population and an osteoporosis clinic population. Arch Osteoporos 14:45. 10.1007/s11657-019-0592-3. 30963310

[34] Ek S, Meyer AC, Sääf M, Hedström M, Modig K (2023) Secondary fracture prevention with osteoporosis medication after a fragility fracture in Sweden remains low despite new guidelines. Arch Osteoporos 18(no. 1):107. 10.1007/s11657-023-01312-z PMC10386957 37516714

[35] Bartosch P, McGuigan FE, Akesson KE (2018) Progression of frailty and prevalence of osteoporosis in a community cohort of older women-a 10-year longitudinal study. Osteoporos Int 29(no. 10):2191–2199. 10.1007/s00198-018-4593-7 PMC6154042 29947868

